# Characterizing the Dynamical Complexity Underlying Meditation

**DOI:** 10.3389/fnsys.2019.00027

**Published:** 2019-07-10

**Authors:** Anira Escrichs, Ana Sanjuán, Selen Atasoy, Ane López-González, César Garrido, Estela Càmara, Gustavo Deco

**Affiliations:** ^1^Computational Neuroscience Group, Department of Information and Communication Technologies, Center for Brain and Cognition, Universitat Pompeu Fabra, Barcelona, Spain; ^2^Cognition and Brain Plasticity Unit, Bellvitge Biomedical Research Institute (IDIBELL), L'Hospitalet de Llobregat, Barcelona, Spain; ^3^Department of Psychiatry, University of Oxford, Oxford, United Kingdom; ^4^Radiology Unit, Hospital Clínic Barcelona, Barcelona, Spain; ^5^Department of Cognition, Development and Educational Psychology, University of Barcelona, Barcelona, Spain; ^6^Institució Catalana de la Recerca i Estudis Avançats, Barcelona, Spain

**Keywords:** ignition, whole-brain, meditation, resting-state, fMRI, integration, dynamical complexity

## Abstract

Over the past 2,500 years, contemplative traditions have explored the nature of the mind using meditation. More recently, neuroimaging research on meditation has revealed differences in brain function and structure in meditators. Nevertheless, the underlying neural mechanisms are still unclear. In order to understand how meditation shapes global activity through the brain, we investigated the spatiotemporal dynamics across the whole-brain functional network using the Intrinsic Ignition Framework. Recent neuroimaging studies have demonstrated that different states of consciousness differ in their underlying dynamical complexity, i.e., how the broadness of communication is elicited and distributed through the brain over time and space. In this work, controls and experienced meditators were scanned using functional magnetic resonance imaging (fMRI) during resting-state and meditation (focused attention on breathing). Our results evidenced that the dynamical complexity underlying meditation shows less complexity than during resting-state in the meditator group but not in the control group. Furthermore, we report that during resting-state, the brain activity of experienced meditators showed higher metastability (i.e., a wider dynamical regime over time) than the one observed in the control group. Overall, these results indicate that the meditation state operates in a different dynamical regime compared to the resting-state.

## 1. Introduction

During the last 2,500 years, contemplative traditions have explored the nature of the mind through self-discipline and self-observation. Meditation per se is not a philosophy or a religious practice, but a method of mental training which enables to cultivate a variety of human abilities, ranging from developing a clearer mind and enhancing attention to cultivating altruistic love and compassion toward other beings (Ricard et al., 2014).

In the last decade, MRI studies exploring the neural correlates of meditation have revealed important insights into how this mental training changes brain function and structure (Brewer et al., 2011; Kilpatrick et al., 2011; Froeliger et al., 2012; Hasenkamp et al., 2012; Taylor et al., 2013; Garrison et al., 2014; Marchand, 2014; Tang et al., 2015; Panda et al., 2016; Kyeong et al., 2017; Mooneyham et al., 2017; Marusak et al., 2018). Yet, little is known about how meditation influences the capability to transmit information across the whole-brain functional network.

Recently, it has been proposed that a brain state can be defined by measuring how the broadness of communication is elicited and distributed through the brain over time, i.e., by characterizing its underlying dynamical complexity (Deco et al., 2017). Investigating the propagation of the neural activity by measuring their dynamical implications (Hutchison et al., 2013) across the whole-brain network may help to explain the fundamental principles of the underlying mechanisms of different brain states (Deco et al., 2011, 2015; Sporns, 2013; Allen et al., 2014). Theoretical methods have been successfully applied to characterize different states of consciousness such as wakefulness, sleep, anesthesia or psychedelic states (Tagliazucchi and Laufs, 2014; Tagliazucchi et al., 2014; Atasoy et al., 2017, 2018; Deco et al., 2017; Jobst et al., 2017).

Here, we investigate the brain's macro-scale mechanisms underlying meditation as well as meditation-induced long-term changes in resting-state using the Intrinsic Ignition Framework (Deco and Kringelbach, 2017; Deco et al., 2017). This data-driven method allows to study the spatiotemporal dynamics across the whole-brain functional network by measuring the effect of naturally occurring local activation events on whole-brain integration.

## 2. Methods

### 2.1. Participants

A total of forty participants were recruited for this experiment. Half of the participants were experienced meditators (mean (SD) age = 39.8 (10.29); education years = 13.6; mean (SD) hours meditation experience = 9526.9 (8619.8); 7 females) and were recruited from Vipassana communities of Barcelona. All of them had a minimum of 1,000 h of meditation experience and confirmed that they maintained daily practice (>1 hour/day). The other half were well-matched control participants with no prior meditation experience (mean (SD) age = 39.75 (10.13); education years= 13.8; 7 females). Participants reported no history of neurological disorder, provided written informed consent, and were compensated for their participation. The study was approved by the Ethics Committee of the Bellvitge Hospital in accordance with the Helsinki Declaration on ethical research.

### 2.2. Resting-State and Meditation fMRI

A total of 450 brain volumes in each condition were analyzed (≈15 min). During rest, participants were asked to look at a fixation cross on the screen, remain as motionless as possible, not to think about anything in particular as well as not to fall asleep. After resting acquisition, all participants were engaged in meditation. Meditators were asked to practice anapanasati meditation (focused attention on breathing). In this type of meditation, subjects try to concentrate all their attention on natural breathing, and when they realize that the mind wanders, they need to recognize it and come back to natural breathing without judgment. Controls were instructed in meditation before being scanned following the instructions as taught by S.N. Goenka (Hart, 1987), who was a Vipassana meditation teacher. Controls confirmed that they understood the procedure after the simulation.

### 2.3. MRI Data Acquisition

MRI images were acquired on a 3T TIM TRIO scanner (Siemens, Erlangen, Germany) using 32-channel receiver coil. The high-resolution T1-weighted images were acquired with 208 slices in the sagittal plane, repetition time (TR) = 1,970 ms, echo time (TE) = 2.34 ms, TI = 1,050 ms, flip angle = 9°, field of view (FOV) = 256 mm, voxel size 1 ×1 ×1 mm. Resting-state and meditation fMRI were performed by a single shot gradient-echo EPI sequence (TR = 2,000 ms; TE = 29 ms; FOV = 240 mm; in-plane resolution 3 mm; 32 transversal slices with thickness = 4 mm; flip angle = 80°).

### 2.4. Preprocessing

Preprocessing was computed using the Data Processing Assistant for Resting-State fMRI (DPARSF) (Chao-Gan and Yu-Feng, 2010). Preprocessing included: manually reorienting T1 and EPI images; discarding the first 10 volumes due to magnetic field inhomogeneities; slice-timing correction; realignment for head motion correction; T1 co-registration to functional image; European regularization segmentation; removal of spurious variance through linear regression: six parameters from the head motion correction, the global mean signal, the white matter signal, and the cerebrospinal fluid signal, CompCor; removal of the linear trend in the time-series; spatial normalization to the Montreal Neurological Institute (MNI); spatial smoothing with 6 mm FWHM Gaussian Kernel; and band-pass temporal filtering (0.01-0.25Hz) (Biswal et al., 1995; Lowe et al., 1998). Finally, we extracted the time-series according to a resting-state atlas of 268 nodes, which ensures the functional homogeneity within each node (Shen et al., 2013).

One meditator was removed due to incidental findings in the MRI session. In addition, 3 controls during meditation and 1 control during rest were excluded due to a head rotation >2 mm or 2°. Moreover, the frame-wise displacement (FD) (Jenkinson et al., 2002) was calculated due to its consideration of voxel-wise differences in motion in its derivation (Yan et al., 2013). Subjects with head motion >2 standard deviations above the group average and movement in more than 25% of time points were excluded from the analysis. FD correction led to the exclusion of 1 control during meditation. Therefore, the final sample of the study included: 19 controls during rest and 16 controls during meditation, 19 meditators during rest and 19 meditators during meditation. After exclusion, no significant differences in terms of age, educational level and gender were observed between groups.

### 2.5. Intrinsic Ignition Framework

**Figure 1:**
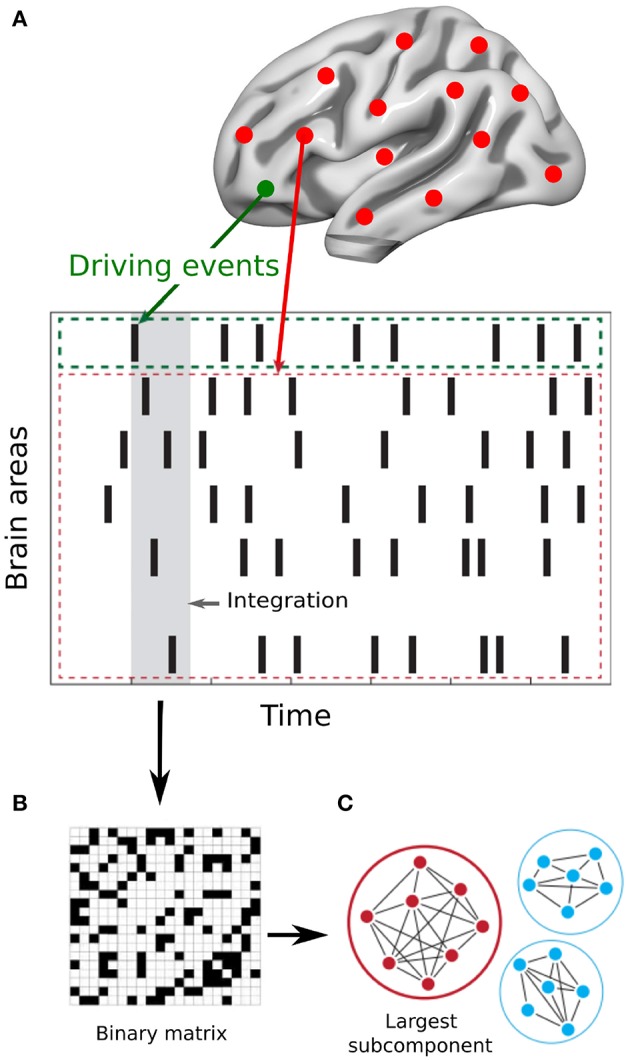
Measuring intrinsic ignition. **(A)** Events were captured applying a threshold method Tagliazucchi et al. (2012) (see green area). For each event elicited (gray area), the activity in the rest of the network was measured in the time-window of 4TR (see red area). **(B)** A binarized matrix was obtained representing the synchronized events in each time window (i.e., when two brain areas have triggered an event) **(C)** Applying the global integration measure Deco et al. (2015), we obtained the largest subcomponent. By repeating the process for each driving event, we calculated the mean and the variability of the Intrinsic-Driven Integration for each brain area across the whole-brain network. Adapted from Deco and Kringelbach (2017).

The Intrinsic Ignition Framework (Deco and Kringelbach, 2017) measures the degree of elicited whole-brain integration of spontaneously occurring events across time. Figure 1 describes the algorithm to obtain the intrinsic integration across events of each brain area. First, the time-series are filtered within the narrowband 0.04–0.07 Hz to avoid artifacts (Glerean et al., 2012). Then, for each brain area, driving events are captured for each timepoint and fixed as a binary signal by transforming the filtered time-series into z-scores, *z*_*i*_(t). A threshold θ is imposed given by the sum of the mean and the standard deviation of the signal in each brain area, such that the binary sequence σ(t) = 1 if *z*_*i*_(t) > θ and is crossing the threshold from below and σ(t) = 0 otherwise (Tagliazucchi et al., 2012). If a brain area has triggered an event (Figure 1A green line) then the integration in the rest of the network is measured within the set time window of 4TR (Figure 1A gray time window). A binary matrix is constructed (Figure 1B) representing the synchronized events in each timepoint (i.e., when two brain areas have triggered an event). Afterwards, the global integration measure (Deco et al., 2015) is defined as the largest component in the binarized connectivity matrix, given by the length of the connected component considered as an adjacency matrix (Figure 1C). Finally, the Intrinsic-Driven Mean Integration (IDMI) is defined as the averaged integration across events, and the variability as the standard deviation of the Intrinsic-Driven Integration. We would like to remark the similitude of our quantitative measure of ignition and the avalanche framework (see, for example, Beggs and Plenz, 2003).

### 2.6. Surrogate Analysis

To ensure that the observed results were not obtained by chance, we applied a surrogate data testing method. Specifically, we randomly permuted the original timeseries across time and measured the ignition in each spontaneous event on the shuffled data. After repeating the process 50 times, we tested whether the empirical ignition values were significantly higher than the surrogates' ignition values.

### 2.7. Statistical Analyses

Here, we compared the IDMI and the variability values for each group (controls and meditators) between conditions (resting and meditation), and we examined if there were differences between groups in the same condition (resting and meditation). Furthermore, we validated our results by comparing the real conditions vs. the randomized ignition data. To do so, we used a Monte-Carlo permutation method. We randomly shuffled the labels between conditions to obtain two new simulated conditions (10,000 permutations). Then, we evaluated how many times the difference between the simulated conditions was higher than the difference between the real conditions. This is, we computed the *p*-value of the null hypothesis that the two random distributions show higher difference than the real conditions. Additionally, we applied the Bonferroni correction for multiple comparisons.

## 3. Results

### 3.1. Intrinsic-Driven Mean Integration (IDMI)

**Figure 2:**
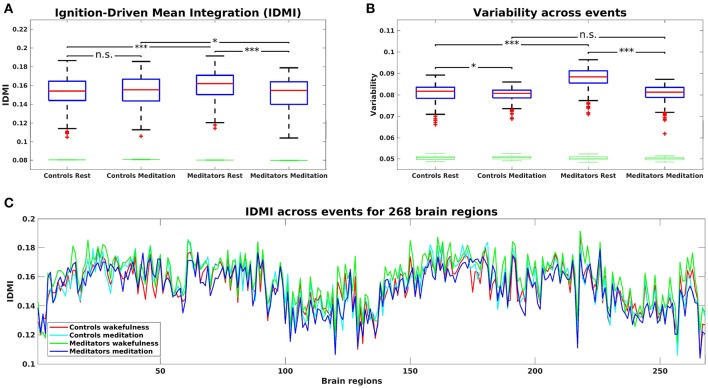
**(A)** Mean of the Intrinsic-Driven Integration (IDMI) for each group during resting-state and meditation state. The IDMI was higher in meditators than in controls during resting-state and lower in meditators during meditation. No significant differences were observed in controls between conditions. Furthermore, we show the box plot from the surrogate IDMI data (on the bottom in green). The randomized data were significantly smaller than the original time-series, showing the robust statistical comparisons. **(B)** Both controls and meditators showed higher local metastability across the whole-brain during resting-state compared to meditation. However, the effect was significantly larger for meditators. Furthermore, the metastability in resting-state was significantly higher for experienced meditators than for controls. *P*-values are based on Monte-Carlo simulation after Bonferroni correction, *p ≤ 0.025, ***p ≤ 0.0005 and n.s represents not significant. **(C)** IDMI across events for each group during resting-state and meditation for 268 brain regions.

Figure 2A shows the IDMI for each group and brain state, while Figure 2C shows the IDMI for each group and each brain area. The IDMI captures the spatial diversity as differences in average intrinsic ignition profiles across the different nodes. The brain activity of meditators during resting-state showed the highest values of the IDMI compared to the control group (*p* < 0.001, Monte-Carlo simulations after Bonferroni correction). Furthermore, this value decreased significantly when meditators were engaged in meditation (*p* < 0.001, Monte-Carlo simulations after Bonferroni correction). In contrast, controls did not show any differences between resting-state and meditation conditions.

### 3.2. Variability of Intrinsic-Driven Integration

Next, we calculated the variability of the Intrinsic-Driven Integration in both states (resting-state and meditation) for each group (controls and meditators). Figure 2B shows the variability for each group and brain state. The variability describes the heterogeneity of each brain area, which is closely connected to its local metastability (Deco and Kringelbach, 2017). Thus, it describes how the local activity in each brain area changes across time. High levels of metastability in a node represent a more dynamic function over time, while lower levels represent greater stability. The brain activity of controls and experienced meditators showed higher functional variability (i.e., metastability) in resting-state than in meditation. Nevertheless, the effect was significantly larger for meditators (*p* < 0.001, Monte-Carlo simulations after Bonferroni correction) than for controls (*p* = 0.022, Monte-Carlo simulations after the Bonferroni correction). Furthermore, the metastability in resting-state was significantly larger for experienced meditators than for controls (*p* < 0.001, Monte-Carlo simulations after the Bonferroni correction).

## 4. Discussion

A growing scientific interest lies in the characterization of the meditation state. Hasenkamp and colleagues (Hasenkamp et al., 2012) captured the interactions between four cognitive phases during meditation, but disregarded the dynamical properties that contain relevant spatiotemporal information. Mooneyham and colleagues applied a dynamical functional connectivity approach dissociating mental states during a meditation scan. The authors reported that after a 6 weeks intervention mindfulness program, subjects spent more time in the state of focused attention and less time in the state of mind-wandering (Mooneyham et al., 2017). In addition, a study that applied graph theoretical analysis (Jao et al., 2016) characterized the degree of the hierarchical organization during meditation. This study revealed that the nodes that had the highest integration degree during rest had the lowest integration degree during meditation, and vice versa. Our work extends these findings by exploring the brain activity during meditation by characterizing the dynamical complexity in terms of how local information is broadcasted across the whole-brain.

Here, we have characterized the dynamical complexity underlying resting-state and meditation in healthy controls and experienced meditators as evidenced by the level of intrinsic ignition. Specifically, in meditators but not in controls, we observed a significant increase of intrinsic ignition during resting-state compared to meditation (Figure 2A). In addition, during resting-state, meditators showed the maximal variability of intrinsic ignition (i.e., metastability) across the whole network, revealing a state of maximum network switching (Figure 2B).

Our results showing an increase of intrinsic ignition during rest compared to meditation are consistent with recent studies on information propagation across the brain. Irrmischer and colleagues found a shift from more complex brain dynamics during rest to a state of reduced information propagation during meditation, importantly, only in meditators (Irrmischer et al., 2018). Furthermore, Gard and colleagues demonstrated using graph theory that yoga and meditation practitioners showed greater network integration than controls during rest (Gard et al., 2014). In addition, the increase of metastability in meditators during resting-state is congruent with the increase of the temporal complexity of oscillations during rest in meditators as observed in the previously mentioned study (Irrmischer et al., 2018). Moreover, studies applying a dynamical functional connectivity approach found that individuals with high trait mindfulness transitioned more frequently between brain states at rest (Lim et al., 2018; Marusak et al., 2018).

To sum up, these results demonstrate that experienced meditators can voluntarily alter their whole-brain dynamics when engaged in a meditative state. Furthermore, expertise in meditation leads to increased ignition and metastability at rest. This means that expert meditators are able to regulate the level of exploration of the dynamical repertoire, restricting it during meditation, and enhancing it during rest.

## Ethics Statement

This study was approved by the Clinical Research Ethics Committee of the Bellvitge University Hospital in accordance with the Declaration of Helsinki. All subjects gave written informed consent to participate in the study.

## Author Contributions

AE and GD designed the study. EC and CG designed the MRI protocol. AE collected the data and wrote the first version of the manuscript. AE and AS pre-processed the fMRI data. AE, AS, and AL-G performed the analyses. AE, AS, GD, and SA interpreted the results. All authors contributed to manuscript revision, read and approved the submitted version.

### Conflict of Interest Statement

The authors declare that the research was conducted in the absence of any commercial or financial relationships that could be construed as a potential conflict of interest.
